# Simulating Illumina metagenomic data with InSilicoSeq

**DOI:** 10.1093/bioinformatics/bty630

**Published:** 2018-07-19

**Authors:** Hadrien Gourlé, Oskar Karlsson-Lindsjö, Juliette Hayer, Erik Bongcam-Rudloff

**Affiliations:** 1 Department of Animal Breeding and Genetics, Swedish University of Agricultural Sciences, SLU-Global Bioinformatics Centre; 2 Department of Molecular Sciences, Swedish University of Agricultural Sciences, Uppsala, Sweden

## Abstract

**Motivation:**

The accurate *in silico* simulation of metagenomic datasets is of great importance for benchmarking bioinformatics tools as well as for experimental design. Users are dependant on large-scale simulation to not only design experiments and new projects but also for accurate estimation of computational needs within a project. Unfortunately, most current read simulators are either not suited for metagenomics, out of date or relatively poorly documented. In this article, we describe InSilicoSeq, a software package to simulate metagenomic Illumina sequencing data. InsilicoSeq has a simple command-line interface and extensive documentation.

**Results:**

InSilicoSeq is implemented in Python and capable of simulating realistic Illumina (meta) genomic data in a parallel fashion with sensible default parameters.

**Availability and implementation:**

Source code and documentation are available under the MIT license at https://github.com/HadrienG/InSilicoSeq and https://insilicoseq.readthedocs.io/.

**Supplementary information:**

Supplementary data are available at *Bioinformatics* online.

## 1 Introduction

With the release of a growing number of bioinformatics tools, it has become challenging to know which tool performs best or is best suited for a particular experiment. The simulation of genomics and metagenomics data holds a prominent role both in the planning of an experiment and the development of new methods. On the contrary to real data, simulated data can be produced with controlled parameters, such as—in the case of metagenomics—the abundance of the species present in a sample. Fixing such parameters allows for benchmarking and testing of new tools in controlled conditions, as well as provides researchers with mock data for testing new tools or pipelines. Such an environment for testing is especially important for fast-growing sub-fields, such as metagenomics (Escalona *et al.*, 2016).

Additionally, simulated data has proven to be very useful in the classroom, where teachers often need mock datasets that are small enough to be analysed quickly and yield meaningful and clear results that are easy to interpret for the students (Halley, 1991).

Surprisingly, only a few such simulation software exist for metagenomics, and the existing solutions are often difficult or inconvenient to use as well as poorly maintained. Here, we describe InSilicoSeq, a software that simulates realistic Illumina reads from (meta)genomes. InSilicoSeq is multi-threaded, well-documented and easily installed via Python’s package manager *pip*. InSilicoSeq aims at making the benchmarking and testing of (meta)genomics software easier.

## 2 Implementation and benchmarks

### 2.1 Implementation and features

InSilicoSeq is written in Python, can accurately model PHRED scores, supports substitution, insertion and deletion errors, as well as insert size distribution and GC bias. InSilicoSeq implements Kernel Density Estimation (KDE) to model base quality and insert size. Briefly, KDE is a non-parametric class of estimators that generally produces a smoother estimation of a distribution (Silverman, 1986) than histograms. Simulation of insertions, deletions, substitutions and GC bias is made empirically and calculated from aligned reads.

In the current release, InSilicoSeq comes with pre-built error models for MiSeq, HiSeq and NovaSeq instruments, but we provide a command to generate error models from any bam file containing aligned reads. The provided error models are calculated from aligned reads in the bam format, generated from three datasets: PRJEB20178 for the MiSeq instrument and public datasets from Illumina Basespace for HiSeq and NovaSeq instruments. The three datasets were assembled with megahit (Li *et al.*, 2015) and the reads were mapped back to the assembly using bowtie2 (Langmead and Salzberg, 2012) with default parameters.

InSilicoSeq being designed for metagenomics, it will generate reads from multiple genomes according to a log-normal abundance distribution per default. Other distributions are built-in, as well as the possibility to provide the software with the exact abundance for each input genome.

### 2.2 Usability

Existing software for simulating metagenomics include MetaSim (Richter *et al.*, 2008), NeSSM (Jia *et al.*, 2013), BEAR (Johnson *et al.*, 2014), FASTQSim (Shcherbina, 2014), GemSim (McElroy *et al.*, 2012), Grinder (Angly *et al.*, 2012), pIRS (Hu *et al.*, 2012) and FunctionSim (Lingling, 2014; https://cals.arizona.edu/∼anling/software/FunctionSIM.htm).

We attempted to install and run all the aforementioned software as well as ART (Huang *et al.*, 2012), a popular single genome simulator; of the nine tested software, only ART, Grinder and pIRS could be installed and run without issues. This is symptomatic of software development in several areas of science, including biology (List *et al.*, 2017; Rother *et al.*, 2012; Wilson *et al.*, 2014) and was one of the main drivers behind the development of InSilicoSeq (Refer to Supplementary Material for more information on usability issues of the other simulators).

### 2.3 Benchmarks

InSilicoSeq can simulate half a million reads in under 10 min (Supplementary Fig. S1) using 4 CPUs and less than 1 G of RAM, and produces more realistic datasets than the other tested simulators. While pIRS ran under 1 min, Grinder took on average more than 13 h to generate half a million reads.

**Fig. 1.**
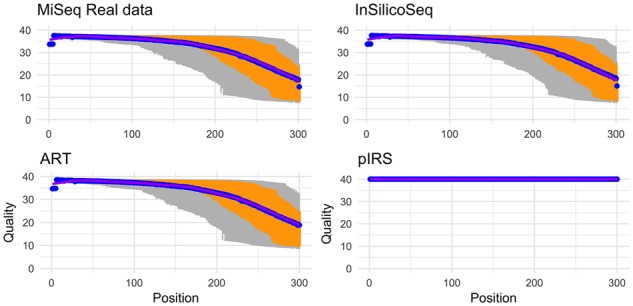
Per Base PHRED score distribution of simulated data (forward reads). The grey lines indicate 10% and 90% quantiles, the orange lines indicate the lower and upper quartiles and the blue dot is the median. InSilicoSeq and ART are the most faithful to the real data, while pIRS assigns a PHRED score of 40 to all bases. For a figure including the forward and reverse reads as well as the qualities from grinder, refer to Supplementary Figures S2 and S3

Figure 1 shows the per-base quality distribution of forward reads simulated with InSilcioSeq, ART and pIRS compared to real data. InSilicoSeq and ART model very closely the base quality of the MiSeq dataset, while pIRS reports all the bases with a PHRED score of 40. For a figure including Grinder as well as reverse reads, refer to Supplementary Figure S2.

One difficult part of generating realistic Illumina data is generating low-quality sequences. InSilicoSeq models this by clustering the sequences by mean quality before modelling the per-base quality distribution. Supplementary Figure S2 shows that our approach out-performs ART, grinder and pIRS: InSilicoSeq is the only simulator to produce low-quality sequences with a mean quality below 20.

## 3 Conclusion

We developed a simulator that is free, open-source, well-tested, easy to install, has sufficient documentation and consists of a unified command (iss). InSilicoSeq produces realistic Illumina data with errors models based on recent Illumina machines and chemistry. New models can be produced from bam files in less than an hour, making it easy to keep them up to date. InSilicoSeq produces more realistic data than existing metagenomics simulation methods and is useful for planning experiments and benchmarking new methods.

## Funding

This work was supported by the Swedish Research Council, grant number 2015-03443_VR. 

*Conflict of Interest*: none declared.

## Supplementary Material

Supplementary Material
