# Supplementary material for: Simulating Illumina metagenomic data with InSilicoSeq
Source: Bioinformatics. 2018 Jul 19;35(3):521–2. doi: 10.1093/bioinformatics/bty630 (PMC6361232; doi:10.1093/bioinformatics/bty630)
Supplement: Supplementary Material [file bty630_supplementary_material.pdf]

Simulating Illumina Metagenomic Data with InSilicoSeq

Supplementary material

Supplementary Figure 1

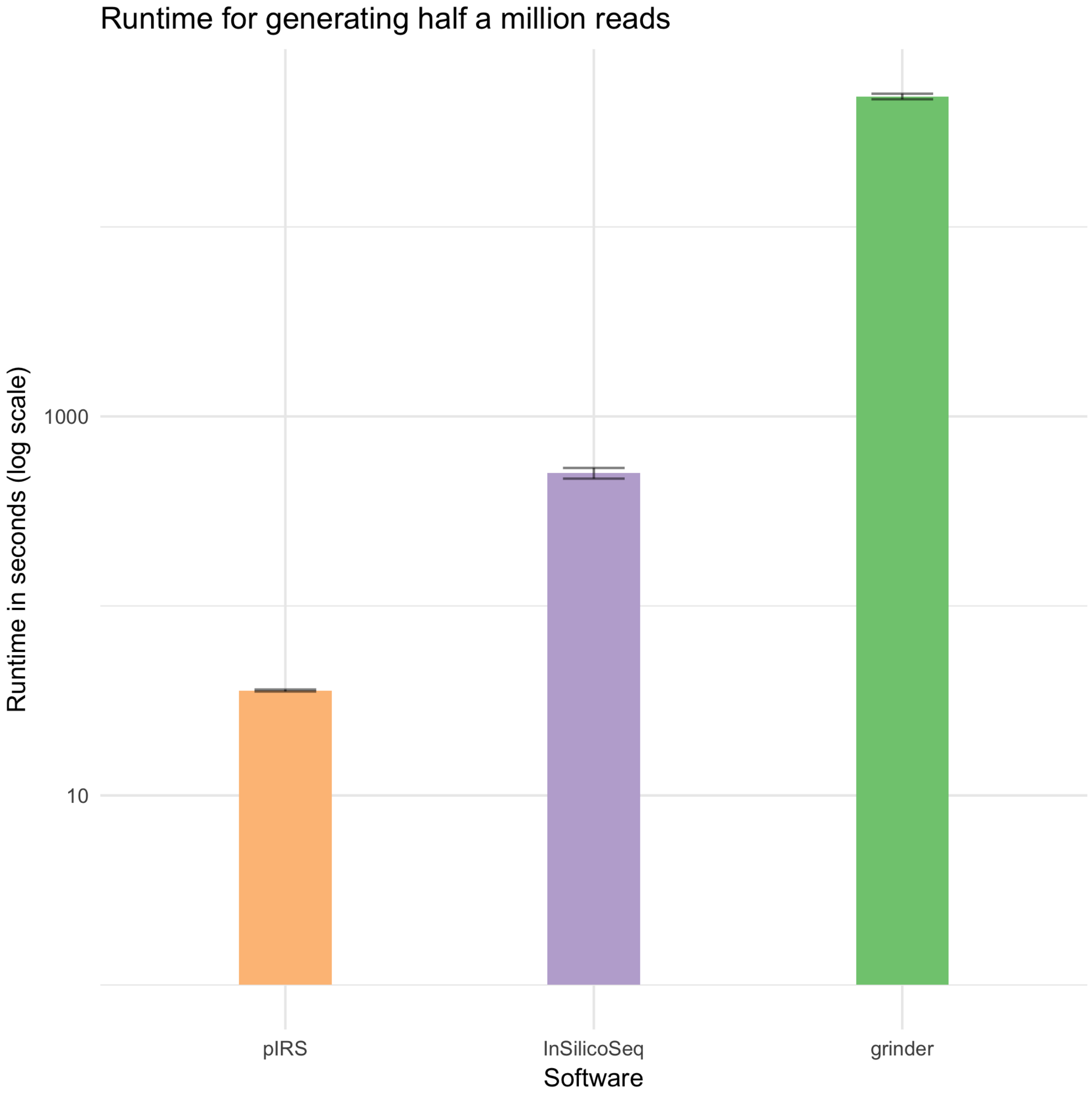

Suppl. Figure 1 shows the runtime of pIRS, InsilicoSeq and Grinder (average over ten runs, log-scale) for simulating half a million under 10 minutes (Pirs in under 1 minute) while grinder took more than 13 hours for each of the ten iterations. Memory usage was measured but is not shown since all three simulators used less than 1G of RAM.

Supplementary Figure 2

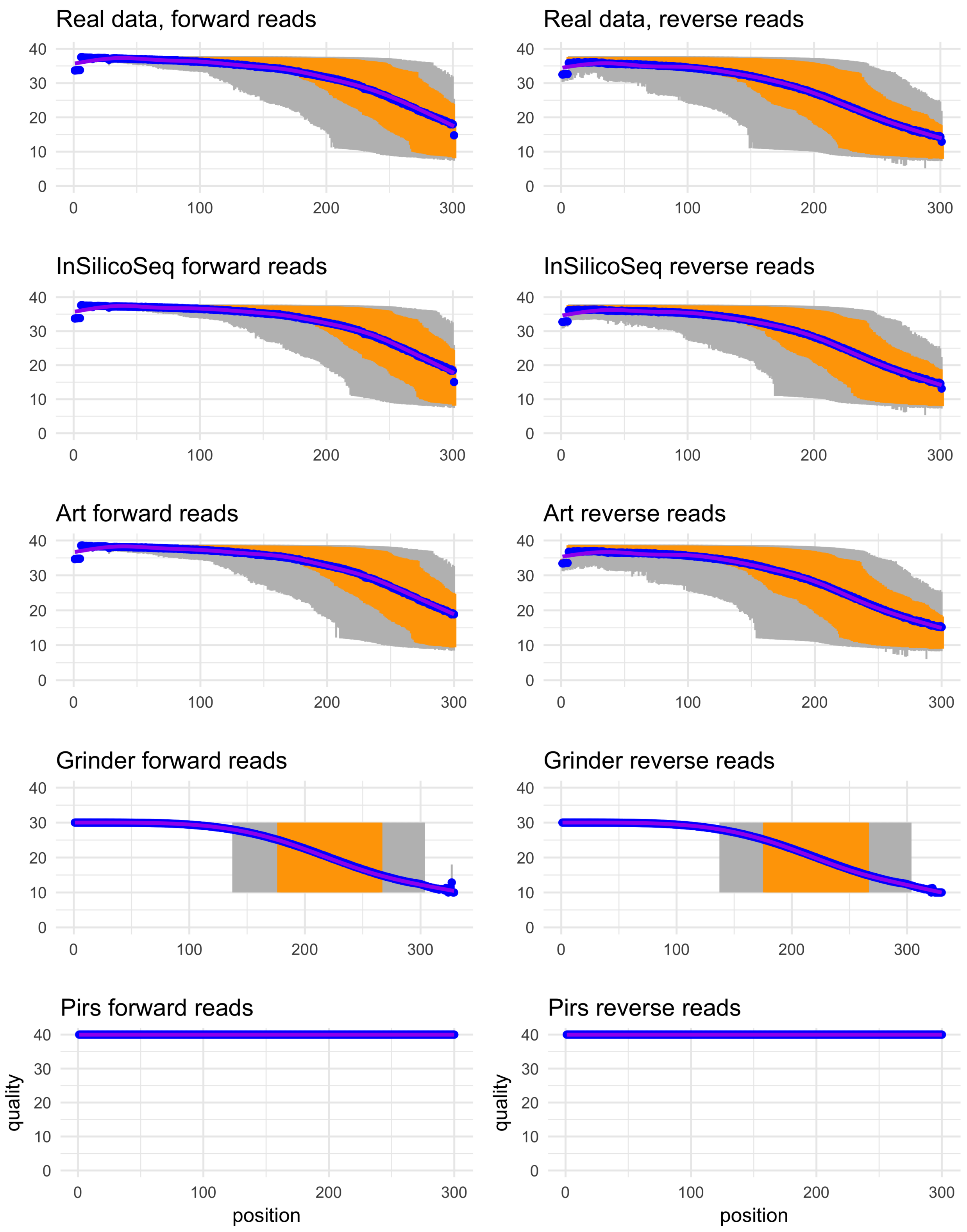

Per Base PHRED score distribution of simulated data (forward and reverse reads). The grey lines indicate

10% and 90% quantiles, the orange lines indicate the lower and upper quartiles and the blue dot is the median. InSilicoSeq and ART are the most faithful to real data, while grinder only generates qualities of either “low” or “high” (Q10 and Q30 in our benchmarks). pIRS only reports Q40.

Supplementary Figure 3

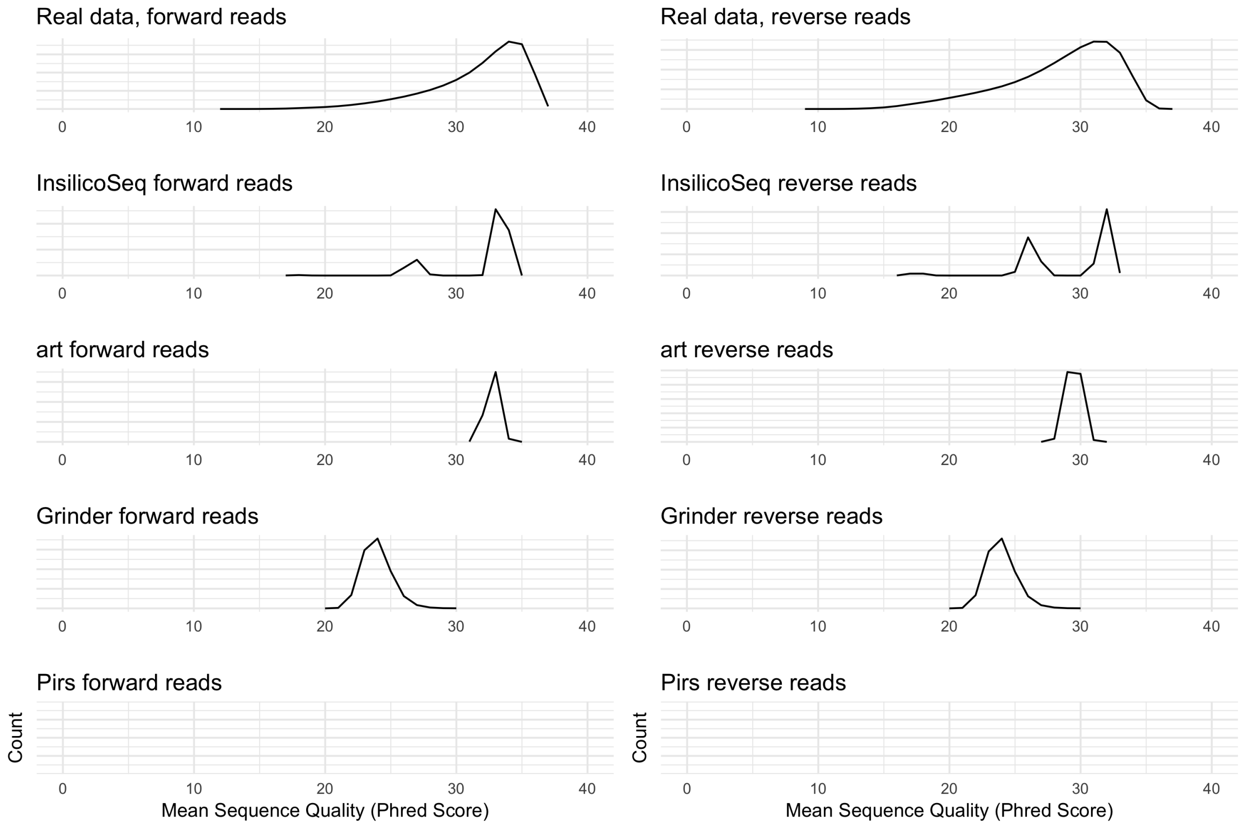

Suppl. figure 3 illustrates the per sequence PHRED score distribution of real and simulated datasets. InSilicoSeq is the only simulator that manages to simulate low-quality sequences.

Supplementary table 1

Software	Open Source	Dependency management	CLI	One command
**InSilicoSeq**	**yes**	**yes**	**yes**	**yes**
MetaSim	no	no	**yes**	**yes**
FunctionSIM	no	no	no	**yes**
NeSSM	no	no	**yes**	no
BEAR	**yes**	no	**yes**	no
FASTQSim	**yes**	no	**yes**	no
GemSim	**yes**	no	**yes**	no
Grinder	**yes**	**yes**	**yes**	**yes**
pIRS	**yes**	no	**yes**	no

Suppl. table 1 highlights issues inherent to academic software development. **Open source** software is important in a research context where researchers tend to build upon each other discoveries, methods and tools. **Dependency management** means that software is available to install by the means of a package manager that will handle dependency resolution for the user. **CLI** means that the software is usable using the command line, which is crucial for automation. Finally, **One command** means that all the functionalities of the software is available under only one command (such as `iss` for InSilicoSeq). While not primordial, it is however important that the pieces of such specialised software do not have generic names such as `split_reads.py` which could create PATH conflicts for the users.

Supplementary table 2

Software	Installation	Recent pre-built models	model building	read generation
InSilicoSeq	**yes**	**yes**	**yes**	**yes**
MetaSim	no	no	no	no
FunctionSIM	no	no	no	no
NeSSM	no	no	no	no
BEAR	**yes**	no	no	no
FASTQSim	**yes**	no	no	no
GemSim	**yes**	no	no	no
Grinder	**yes**	no	no	**yes**
pIRS	**yes**	no	**yes**	**yes**

Suppl. Table 2 is summary of issues encountered with the different simulators tested. The two last columns, "model building" and "read generation" indicate wether we managed to run the software successfully or not.

General comments about the usability of the aforementioned simulators

1. MetaSim

Disadvantages:

- Last updated in 2009
- Not open source
- Requires a graphical install
- Only outputs fasta files, does not model quality.

2. FunctionSIM

Disadvantages:

- Can only model Illumina GA IIx or 454
- Not open source
- No license information.

3. NeSSM

Disadvantages:

- Academic license
- NeSSM consists in a collection of scripts with generic names that could conflict with others in the PATH

Installation issues:

- `complete_update_step.pl` first fails to download data from the ncbi FTP due to the recent change in structure:

```
Resolving ftp.ncbi.nih.gov (ftp.ncbi.nih.gov)... 130.14.250.12, 2607:f220:41e:250::13
Connecting to ftp.ncbi.nih.gov (ftp.ncbi.nih.gov)|130.14.250.12|:21... connected.
Logging in as anonymous ... Logged in!
==> SYST ... done.    ==> PWD ... done.
==> TYPE I ... done.  ==> CWD (1) /genomes/Bacteria ...
No such directory ‘genomes/Bacteria’.
```

then fails at downloading the fungi due to a missing script in NeSSM

```
Can't open perl script "dl_fungi.pl": No such file or directory
usage: perl mk_index.pl whole_pathway_datebase_dir/ output_dir/
```

4. BEAR

Disadvantages:

- academic license
- collection of scripts with generic names that may conflict with others in the PATH
- not maintained anymore

Issues while running:

- failed to run their modified version of drisee

5. FASTQSim

Disadvantages:

- collection of scripts with generic names that may conflict with others in the PATH
- have to be in the fastqsim install directory to execute it
- KeyboardInterrupt does not work and various errors are not properly caught.
- needs a background host dataset hence does not work for environmental samples

6. GemSim

Disadvantages:

- only pre-built Illumina GAII models

Issues while running:

- The model building script failed after 7 hours with the following traceback

```
Traceback (most recent call last):
  File "/home/hadrien/workspace/GemSIM_v1.6/GemReads.py", line 834, in <module>
    main(sys.argv[1:])
  File "/home/hadrien/workspace/GemSIM_v1.6/GemReads.py", line 755, in main
    refDict,genDict,comDict,refList=getMet(direct,genDirect, abund)
  File "/home/hadrien/workspace/GemSIM_v1.6/GemReads.py", line 111, in getMet
    val=float(parts[1])
```

```
IndexError: list index out of range
```

- Read generation Using the provided GAII model failed without any error message

7. Grinder

Disadvantages:

- no error models, the user has to know error rates
- output pairs in the same file

8. pIRS

Disadvantages:

- compile to `pirs` but extra scripts with generic names
- paths to prebuilt profiles hardcoded to `/usr/local/share/pirs/` and no instruction for modifying the installation behavior.
